# Effects of Tiliroside and Lisuride Co-Treatment on the PI3K/Akt Signal Pathway: Modulating Neuroinflammation and Apoptosis in Parkinson’s Disease

**DOI:** 10.3390/biomedicines11102735

**Published:** 2023-10-09

**Authors:** Faisal K. Alkholifi, Sushma Devi, Mohammed F. Aldawsari, Ahmed I. Foudah, Mohammed H. Alqarni, Mohamad Ayman Salkini, Sherouk Hussein Sweilam

**Affiliations:** 1 Department of Pharmacology & Toxicology, College of Pharmacy, Prince Sattam Bin Abdulaziz University, Al Kharj 11942, Saudi Arabia; 2 Chitkara College of Pharmacy, Chitkara University, Rajpura 140401, India; 3 Department of Pharmaceutics, College of Pharmacy, Prince Sattam Bin Abdulaziz University, Al Kharj 11942, Saudi Arabia; 4 Department of Pharmacognosy, College of Pharmacy, Prince Sattam Bin Abdulaziz University, Al Kharj 11942, Saudi Arabia; a.foudah@psau.edu.sa (A.I.F.); m.alqarni@psau.edu.sa (M.H.A.);; 5 Department of Pharmacognosy, Faculty of Pharmacy, Egyptian Russian University, Badr City 11829, Egypt

**Keywords:** Tiliroside, Lisuride, MPTP, Parkinson’s disease, pAkt/Akt, Bcl-2, TNF-α, IL-1β

## Abstract

Researchers are actively exploring potential bioactive compounds to enhance the effectiveness of Lisuride (Lis) in treating Parkinson’s disease (PD) over the long term, aiming to mitigate the serious side effects associated with its extended use. A recent study found that combining the dietary flavonoid Tiliroside (Til) with Lis has potential anti-Parkinson’s benefits. The study showed significant improvements in PD symptoms induced by 1-Methyl-4-phenyl-1,2,3,6-tetrahydropyridine (MPTP) when Til and Lis were given together, based on various behavioral tests. This combined treatment significantly improved motor function and protected dopaminergic neurons in rats with PD induced by MPTP. It also activated important molecular pathways related to cell survival and apoptosis control, as indicated by the increased pAkt/Akt ratio. Til and Lis together increased B-cell lymphoma 2 (Bcl-2), decreased caspase 3 activity, and prevented brain cell decay. Co-administration also reduced tumor necrosis factor alpha (TNF-α) and Interleukin-1 (IL-1). Antioxidant markers such as superoxide dismutase (SOD), catalase, and reduced glutathione significantly improved compared to the MPTP-induced control group. This study shows that using Til and Lis together effectively treats MPTP-induced PD in rats, yielding results comparable to an 8 mg/kg dose of levodopa, highlighting their potential as promising Parkinson’s treatments.

## 1. Introduction

The complex nature of Parkinson’s disease (PD) affects cognition, psychology, and society. Loss of dopaminergic neurons in the substantia nigra pars compacta causes PD [1]. The neurotoxin 1-Methyl-4-phenyl-1,2,3,6-tetrahydropyridine (MPTP) illuminates PD processes. MPTP specifically targets dopaminergic neurons in the substantia nigra pars compacta by inhibiting mitochondrial complex I. This poisonous substance mimics idiopathic PD symptoms, helping to explain its etiology [2]. MPTP forms mitochondria-damaging 1-methyl-4-phenylpyridinium (MPP+). By increasing intracellular reactive oxygen species, MPP+ causes dopaminergic neuron death. The distinctive attributes of both MPTP and its metabolite MPP+ render them valuable instruments within experimental models, facilitating the investigation of PD in controlled laboratory environments, both in vitro and in vivo [3,4,5]. By harnessing these models, researchers can acquire insights into the intricate mechanisms that underlie PD and formulate potential interventions or therapies to alleviate its impact. Consequently, the study of MPTP and MPP+ contributes significantly to the advancement of our comprehension of PD and potentially lays the groundwork for future treatments.

The etiology of PD processes may be brought on by a variety of factors, such as reactive oxygen species, oxidative stress, neuroinflammation, and mitochondrial dysfunction. Despite the lack of knowledge on the precise causes of PD, these pathways have a role in the disease’s development and progression [6]. Nuclear factor kappa B (NF-κB) and the neuroinflammatory response are both involved in this pathway. When NF-κB is activated, it binds to certain response areas on genes linked to inflammation. NF-κB may stimulate gene transcription via this binding, which results in the creation of pro-inflammatory proteins such tumor necrosis factor alpha (TNF-α), Interleukin-1 (IL-1), and caspase. However, these are not the only elements that influence NF-κB. NF-κB controls the creation of pro-inflammatory substances by attaching to gene promoter response elements, hence sustaining neuroinflammation [7].

The investigation of neuroinflammatory pathways involving MPTP, MPP+, and related factors holds significant importance for PD research. Bridging the gap between neurotoxicity and neurodegeneration, this integrated approach unveils potential therapeutic targets and strategies. Moreover, the phosphatidylinositol 3-kinases-protein kinase B (PI3K-AKT) signaling pathway plays a pivotal role by regulating signal transduction, cell proliferation, apoptosis, and metabolism. This pathway’s impact on neurotoxicity and neuron survival has been established. Emerging evidence highlights that natural products can activate the PI3K-AKT pathway, demonstrating neuroprotective effects and offering prospects for discovering novel therapeutic interventions for PD [8].

However, despite the advances in understanding PD’s pathogenesis and the availability of symptomatic treatments such as levodopa, the disease’s complexity remains a challenge. Levodopa, while effective in managing symptoms by replenishing dopamine levels, lacks the ability to halt or slow down disease progression [9]. This limitation underscores the need for alternative strategies that comprehensively address both symptom relief and long-term disease advancement [10]. The ideal approach for anti-PD drugs should encompass symptom alleviation, neurodegeneration prevention, and restoration of dopaminergic neurons [11].

The current medical landscape is marked by the utilization of Lisuride (Lis), an isolysergic acid derivative, to effectively address an array of conditions such as migraine, cluster headache, acromegaly, hyperprolactinemia, and PD [12]. Functioning as a dopamine receptor agonist, Lis demonstrates antagonistic properties towards the 5-hydroxytryptamine2B (5-HT2B) receptor. Although levodopa, a dopamine precursor, has traditionally served as the primary therapeutic approach for alleviating PD symptoms for an extended period, spanning over three decades [13], the prolonged administration of dopamine replacement therapy has been associated with incapacitating adverse effects. Notably, complications such as levodopa and dopamine-agonist-induced dyskinesia can arise. In light of these challenges, alternative treatment strategies for PD have emerged, incorporating diverse direct-acting dopamine receptor agonists such as apomorphine, pergolide, cabergoline, ropinirole, pramipexole, bromocriptine, and the aforementioned Lis [14,15].

Existing research on flavonoids in neurological disorders focuses on their general neuroprotective potential, lacking emphasis on PD’s unique complexities [16]. This results in a research gap regarding how flavonoids [17,18], particularly Tiliroside (Til), could be leveraged for innovative PD treatments. The identified properties of Til, including its anti-inflammatory, antioxidant, and neuroprotective effects [19], highlight its potential as a therapeutic agent for various biological processes. However, the extent to which Til can effectively mitigate the intricate interplay of symptoms relief, neurodegeneration prevention, and dopaminergic neuron restoration in PD remains largely uncharted [20]. Furthermore, as indicated by a study, Til has been observed to possess the capability of activating both AMP-activated protein kinase and peroxisome proliferator-activated receptor α in mice afflicted with obesity and diabetes [21]. While it has been demonstrated that Til can inhibit neuroinflammation in activated BV2 microglia through the TRAF-6/NF-κB/p38 pathway [22], its broader impact on the complex pathogenesis of PD, encompassing dopaminergic dysfunction and disease progression, requires deeper investigation. Thus, there is a need to delve into the precise mechanisms underlying Til’s potential to comprehensively address symptom relief, neuroprotection, and neuronal restoration in PD.

**Scheme 1:**
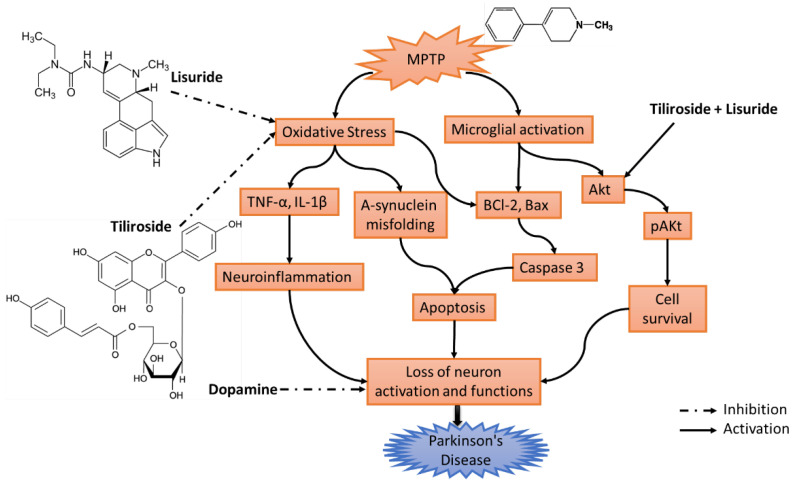
Hypothesis of the proposed work. MPTP can trigger oxidative stress and activates microglial cells. The activation of microglia and oxidative stress can lead to the upregulation of pro-inflammatory molecules such as TNF-alpha, B-cell lymphoma 2 (Bcl-2), Bax, etc. These inflammatory processes can further contribute to neuronal damage and the death of dopaminergic neurons. Meanwhile, decreased AKT activity may render neurons less capable of surviving and coping with the stressors encountered in PD. Til+Lis showed activation and inhibition on these pathways. Abbreviations: MPTP—1-Methyl-4-phenyl-1,2,3,6-tetrahydropyridine; TNF-α—tumor necrosis factor alpha; IL-1β—Interleukin-1 beta; BCl-2—B-cell lymphoma 2; Bax—Bcl-2-associated X protein; Akt—Ak strain transforming; pAKt—phosphorylated Akt.

Given the existing gaps in research, our study was designed to delve into the potential synergies between Til administered at dosages of 10 and 20 mg/kg/day and Lis administered at 0.1 mg/kg/day in mitigating symptoms associated with PD. The primary objective of this investigation was to assess the combined treatment approach involving Til and Lis using animal models of PD. Til, recognized for its anti-inflammatory and antioxidant attributes, and Lis, a dopamine agonist, were chosen due to their potential in ameliorating PD symptoms. The investigation aimed to determine the effects of varying Til doses in conjunction with a consistent dose of Lis on the PD symptoms. The hypothesis of the current manuscript is shown in Scheme 1.

## 2. Materials and Methods

### 2.1. Chemicals

Til and Lis were given as samples by Myoford Pharma in Solan, India. MPTP hydrochloride (MPTP-HCl) was obtained from Sigma Aldrich, St. Louis, MO, USA. Both Til and Lis were solubilized in 80% ethanol. Thiobarbituric Acid Reactive Substances (TBARS) assay kit (E-BC-K298-M) was obtained from Elabscience, Houston, TX, USA, and Superoxide Dismutase (SOD) activity assay kit (CS0009) and Glutathione (GSH) assay kit (MAK364) were obtained from Sigma-Aldrich Solutions, Darmstadt, Germany. Catalase assay kit was purchased from MyBioSource Inc., San Diego, CA, USA. Rat Caspase 3 ELISA Kit (E-EL-R0160) and Rat IL-1β ELISA Kit (E-EL-R0012) were obtained from Elabscience, USA. Rat Bcl-2 ELISA Kit (E-EL-R0096) was obtained from Elabscience. TNF-α ELISA kit (MBS825075) and Nuclear Factor Kappa B ELISA Kit (MBS453975) were obtained from R&D Systems, Minneapolis, MN, USA. Anti-Akt Antibody (ab8805) was obtained from Abcam Inc., Cambridge, MA, USA. All other reagents used were of analytical grade.

### 2.2. Animals

Male Wistar rats, aged 7 to 8 weeks and weighing between 210 g and 228 g, were obtained from the National Institute of Pharmaceutical Education and Science in Mohali, India. Upon arrival, a one-week adaptation phase was initiated for the rats, during which they were exposed to consistent temperature conditions and a 12 h light–dark cycle. Their well-being was actively monitored throughout this period. The study was granted ethical clearance (approval No.: SSP/IAEC/18/04) by the Institutional Animal Ethics Committee for the execution of animal experiments. All animal care procedures strictly adhered to guidelines set by CPCSEA, Ministry of Forests and Climate Change, Government of India, as well as those outlined by the Indian National Science Academy. A two-week acclimatization period was implemented before commencing any experimental interventions, allowing the rats to become familiar with their new environment. Throughout the duration of the research, the rats were provided with a nutritionally balanced pellet diet (Ashirwad Industries) and were given unrestricted access to clean water, in accordance with CPCSEA regulations.

### 2.3. Experimental Design and Drug Treatments

**Scheme 2:**
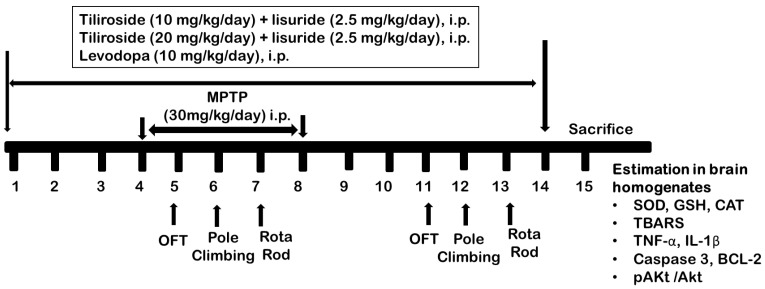
Experimental design.

Scheme 2 is used to illustrate the series of steps that were involved in conducting an experiment. The animals’ vital signs and weight were regularly assessed throughout the entire duration of the trial, ensuring that specified protocols for animal care were followed. A cohort of 40 rats in total was divided into groups, each consisting of eight members, based on predetermined criteria.

Group I: The control group, which consisted of rats receiving a daily dose of the vehicle (0.9% saline) for 14 consecutive days. The vehicle used in this experiment contained no active ingredients and served as a baseline to compare with other experimental groups.

Group II: MPTP control; rats were initially administered the vehicle (0.9% saline) for the first 3 days, followed by daily administration of MPTP at a dose of 30 mg/kg/day, i.p., from day 4 to day 8. Thereafter, no additional treatments were provided.

Group III: Til 10 + Lis; the group received 10mg/kg, i.p., Tiliroside and 2.5 mg/kg, i.p., Lisuride dissolved in vehicle from day 1 to 14. From the 4th to the 8th day, the rats received 30 mg/kg, i.p., MPTP as in group II.

Group IV: Til 20 + Lis; this group received 20 mg/kg, i.p., Tiliroside and 2.5 mg/kg, i.p., Lisuride dissolved in vehicle from day 1 to 14. From the 4th to the 8th day, the rats received 30 mg/kg MPTP as in group II.

Group V: Levodopa; rats were administered levodopa (8 mg/kg/day, i.p.) from day 1 to 14, followed by MPTP (30 mg/kg/day, i.p.) from day 4 to day 8 as in group II.

The initial behavioral tests in animals were studied on days 5, 6, and 7. The final behavioral assessments were on days 11, 12, and 13. On day 15, the animals were scarified, and their brains were isolated for biochemical analysis [23,24].

### 2.4. Behavioral Tests

#### 2.4.1. Open-Field Test

To assess the rats’ natural motor function and their tendency for exploration, a methodology known as the open-field test (OFT) was employed. Prior to conducting this assessment, it was essential to familiarize the rats with their environment in accordance with protocols previously established by Foudah et al., 2022 [25]. Throughout the experiment, each individual rat was positioned at the midpoint of the area and observed for 5 min. The parameters assessed included line crossings, rearing behavior, grooming activity, and immobility time [25].

#### 2.4.2. Pole-Climbing Test

The pole-climbing assessment was conducted with a divided 50 cm pole, where each section measured 25 cm. A separate point had been marked at the center of the pole, and a small ball covered in gauze had been positioned on top to avoid any slipping. The rat was released onto the pole and timed until the designated line was reached. A score was assigned based on performance: taking longer than 6 s resulted in one point, completing the task in 3–6 s earned two points, and finishing in less than three seconds garnered three points [26].

#### 2.4.3. Rotarod Test

To evaluate the strength of movement, the rotarod test was performed. The experimental setup involved a rotarod apparatus that started rotating at an initial speed of 4 revolutions per minute (rpm) and gradually accelerated to reach a maximum speed of 40 rpm within five minutes. Following this, there was a resting interval lasting for another five minutes. Each rat underwent three trials during each designated time point in order to obtain reliable data for analysis [27].

### 2.5. Preparation of Tissue for Biochemical Estimation

After the behavioral assessments, rodents were anaesthetized using an intraperitoneal administration of sodium pentobarbital (40 mg/kg) and sacrificed by decapitation. One portion of their brains was preserved in a 10% formaldehyde solution to facilitate subsequent morphological analysis. Meanwhile, the other half was promptly frozen using dry ice and stored at −80 °C until it could be subjected to biochemical analyses [28].

### 2.6. Estimation of Catalase Level

The detection of catalase activity in biological samples was accomplished using the Catalase Kit provided by MyBioSource Inc., San Diego, CA, USA. This comprehensive kit consisted of 96-well plate, a catalase standard with a concentration of 100 Units/mL, substrate solution, and other necessary assay components. The measurement was performed according to kit protocol. At the end of reaction, a colored product was measured at a wavelength of 560 nm. Higher levels of catalase in the samples resulted in a decrease in hydrogen peroxide concentration and a corresponding decrease in the formation of the pink product [29].

### 2.7. Estimation of Superoxide Dismutase Level

In order to evaluate the activity of superoxide dismutase, a series of procedures was carried out according to SOD activity assay kit from Sigma-Aldrich Solutions, Germany. At first, a 50 µL volume from the homogenate sample was mixed with precise amounts of distilled water, sodium pyrophosphate buffer, post-mitochondrial supernatant, nitroblue tetrazolium, and nicotinamide adenine dinucleotide phosphate. The solution was subsequently mixed with potassium phosphate buffer until the total volume reached approximately 50 milliliters. To terminate the reaction, an additional amount of potassium phosphate buffer was added. Optic densities were employed to evaluate the efficacy levels in samples, and concentration was measured in milligrams [30].

### 2.8. Estimation of Reduced Glutathione Level

The GSH assay was performed using kits from Sigma-Aldrich Solutions, Germany. The procedure was performed according to manufacturer’s protocols and by measuring the absorbance values, determining the microgram per milligram of protein that represents the concentration of reduced glutathione [31].

### 2.9. Thiobarbituric Acid Reactive Substances (TBARS) Assay (Estimation of Lipid Peroxidation)

In order to quantify the amount of lipid peroxidation, TBARS assay was performed. To accomplish this, a series of steps were performed as per manufacturer’s manual (Elabscience, USA). The absorbance value was measured at a wavelength of 532 nm and compared against reference standards [32].

### 2.10. Estimation of TNF-α

The ELISA kit from R&D Systems, Minneapolis, MN, USA was used to measure the levels of TNF-α. To quantify the concentration expressed as pg/mL, optical density absorbance readings were obtained at a wavelength value of 450 nm using a microplate reader device. These measurements serve as key indicators for determining sample concentrations [33].

### 2.11. Estimation of IL-1β

For the estimation of IL-1β level, ELISA Kit, Elabscience, USA, was used. The kit instructions’ experimental procedure was followed precisely. The absorbance of the plate was immediately measured at a wavelength of 450 nm using an appropriate instrument. The obtained data were then processed to determine the concentration in pg/mL units [34].

### 2.12. Estimation of NF-κB

To carry out the experiment, the NF-κB level was estimated using NF-κB ELISA Kit obtained from R&D Systems located in Minneapolis, MN, USA. The manufacturer’s instructions were carefully followed. A microplate reader measured results immediately by capturing readings at an absorbance wavelength set to 450 nm. The final outcomes were represented as ng/mL [35].

### 2.13. Estimation of BCl-2

To determine the levels of BCL2, the Rat Bcl-2 ELISA Kit, Elabscience, USA, was used. Adhering to the guidelines stipulated in the manual, the optical density readings were measured at a wavelength of 450 nm, and the quantified results were expressed as ng/mL [36].

### 2.14. Estimation of Caspase 3

To assess the levels of caspase 3, ELISA Kit (E-EL-R0160) Elabscience, USA, was used. To ensure precise measurements, the protocol outlined in the kit manual was followed. The absorbance reading took place at a wavelength of 450 nm, and the results were presented as ng/mL [37].

### 2.15. Estimation of pAkt/Akt Using Western Blotting

To estimate the pAkt/Akt protein analysis, the brain supernatant homogenate was transferred to a nitrocellulose membrane through electrophoresis following SDS-PAGE. To block non-specific binding, the nitrocellulose membrane was incubated at room temperature for 2 h. Subsequently, polyclonal antibodies targeting anti-Akt Antibody (ab8805) from Abcam Inc., Cambridge, MA, USA, were added and left overnight at 4 °C for antibody–antigen interaction on the membrane. Afterward, the nitrocellulose membranes underwent thorough washing in a solution lasting for about 30 min to remove any unbound antibodies or other impurities. For identification purposes of immunoreactive lanes, BandScan software version 5.0 was utilized followed by signal amplification using chemiluminescence before digitalization [38].

### 2.16. Statistical Analysis

All values were presented as means ± standard error of the mean and visualized using Microsoft Excel 13. The statistical analysis was performed using GraphPad Prism 7 software. For the assessment of biochemical, behavioral testing, and Western blot analysis results, a one-way analysis of variance followed by Tukey’s multiple comparison test was employed. Differences were considered statistically significant if the *p*-value was less than 0.05.

## 3. Results

### 3.1. Effect of Co-Treatment of Til and Lis on an Open-Field Test

The open-field test evaluates several parameters, including line crossings, rearing behavior, grooming activity, and immobility time, as given in Table 1. The provided table presents data on various behavioral measures for different groups across different days of observation. The table consists of four groups: Normal, MPTP control, Til 10 + Lis, Til 20 + Lis, and Levodopa. The behaviors being measured are line crossing, rearing, grooming, and immobility. The normal group serves as a baseline, and their measurements are provided as means along with their standard deviations. The values for each parameter suggest the typical or expected behavior for these subjects. On day 5, the normal group exhibited an average of 141.67 line crossings, which increased slightly to 150.83 on day 11. Similarly, the number of rearing behaviors increased from 15.67 on day 5 to 17.67 on day 11 for the normal group. Grooming behaviors increased from 20.33 to 25.33, while immobility decreased from 11.33 to 18.83 between the two time points. The MPTP control group showed considerably lower values across all behaviors on both days compared to the normal group, indicating impaired behavior due to the MPTP treatment. Notably, there was a significant increase in immobility from 194.33 on day 5 to 215.67 on day 11 in the MPTP control group. The groups Til 10 + Lis, Til 20 + Lis, and Levodopa also exhibited changes in their behaviors between the two time points. These groups demonstrated varying degrees of line crossing, rearing, grooming, and immobility. The Til 20 + Lis group, for instance, showed an increase in most behaviors on day 11 compared to day 5. Overall, the table indicates that the different treatment groups and the “MPTP control” group exhibited alterations in behavior over the course of the study, suggesting the impact of treatments on these behavioral measures. The results highlight the potential effects of different interventions and treatments on the observed behaviors, providing insights into the experimental outcomes.

### 3.2. Effect of Co-Treatment of Til and Lis on Pole Climbing, Holding Time, and Latent Time

**Figure 1:**
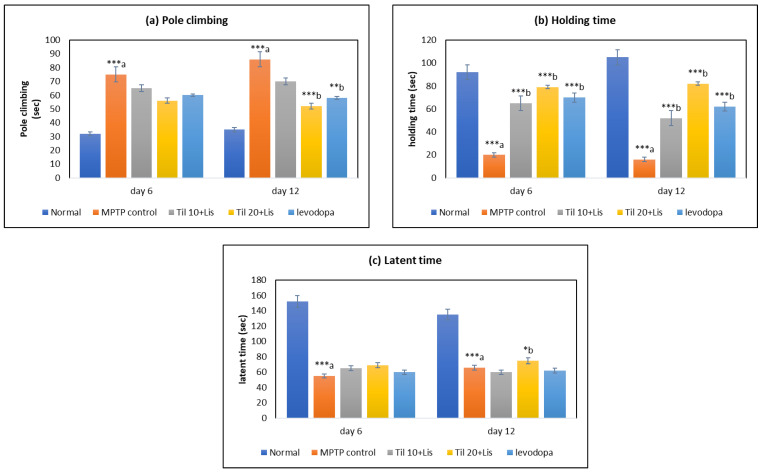
Effect of Til and Lis co-treatment on rats’ behavior in pole climbing: (**a**) pole climbing time (day 6 F value—9.92; day 12 F value—18.48); (**b**) holding time (day 6 F value—61.84; day 12 F value—65.87); (**c**) latent time (day 6 F value—46.73; day 12 F value—40.12). The statistical significance was determined using a threshold of *p*-value (* *p* < 0.05; ** *p* < 0.01; *** *p* < 0.001). “a”—normal vs. MPTP control; “b”—MPTP control vs. treatment groups. Data are expressed as the mean ± S.E.M. (*n* = 6).

Significant alterations in pole climbing time, holding time, and latent time were observed in the MPTP-exposed group of rats compared to the normal control group (*p* < 0.001), as depicted in Figure 1. However, a marked improvement was noted in both pole climbing time held by the rats on day 12 (*p* < 0.01, *p* < 0.001) when Til and Lis were simultaneously administered as a co-treatment. Furthermore, substantial enhancement in locomotor behavior was observed overall on day 6, with even more remarkable improvement seen on day 12. These findings underscore the potential benefits of the combined treatment using Til and Lis for enhancing motor performance within this specific experimental model.

### 3.3. Effect of Co-Treatment of Til and Lis on Rotarod Test

**Figure 2:**
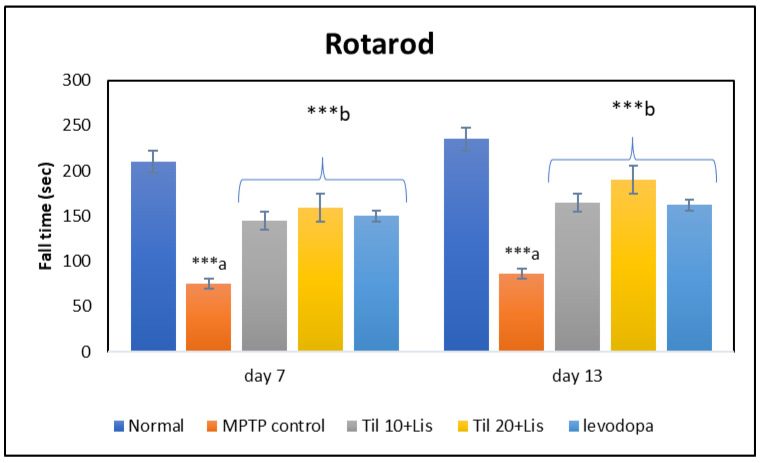
Effect of Til and Lis co-treatment on fall time in rotarod. The statistical significance was determined using a threshold of *p*-value (*** *p* < 0.001) (day 7 F value—53.26; day 13 F value—96.95). “a”—normal vs. MPTP control; “b”—MPTP control vs. treatment groups. Data are expressed as the mean ± S.E.M. (*n* = 6).

The administration of Til and Lis in rotarod tests resulted in a significant increase in the drop time of MPTP-treated rats compared to the control group, as shown in Figure 2. Specifically, the drop time increased from 78.44 ± 3.25 s in the MPTP control group to 212.70 ± 6.27 s (*p* < 0.001) when rats were treated with this combination therapy. This co-treatment approach effectively brought back the fall time closer to that observed in normal rats. It is worth mentioning that when comparing high doses of Til combined with Lis against levodopa treatment alone, better outcomes were consistently observed for this novel combination therapy.

### 3.4. Effect of Co-Treatment of Til and Lis on CAT, SOD, and GSH

**Figure 3:**
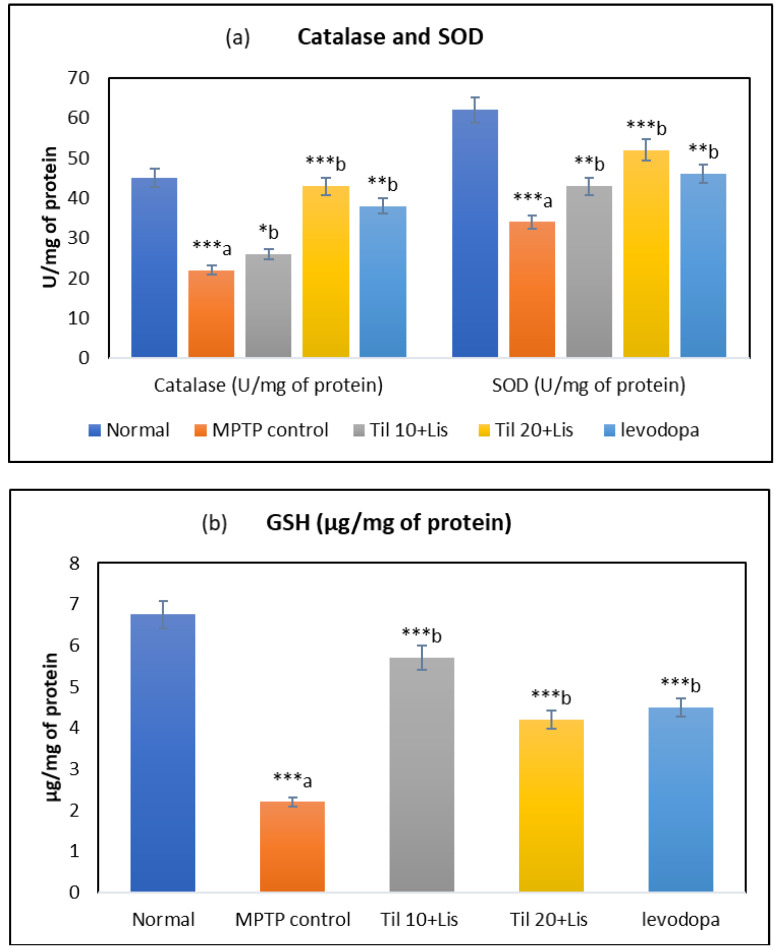
Effect of Til and Lis co-treatment on antioxidant levels. (**a**) Catalase and SOD and (**b**) GSH. The statistical significance was determined using a threshold of *p*-value (* *p* < 0.05; ** *p* < 0.01; *** *p* < 0.001). “a”—normal vs. MPTP control; “b”—MPTP control vs. treatment groups. Data are expressed as the mean ± S.E.M. (*n* = 6). (F value of CAT—7.61; SOD—11.30; GSH—4.03.).

The determination of oxidation levels in this investigation was carried out by analyzing three key components: superoxide dismutase, catalase, and reduced glutathione. A vital role was played by these three elements as part of the body’s natural defense system against oxidative stress, as illustrated in Figure 3. The findings revealed that the activities of SOD and CAT were significantly decreased, along with reduced levels of GSH within the MPTP group compared to normal controls (*p* < 0.001). However, the declines induced by MPTP administration were successfully mitigated by the administration of Til and Lis together; a remarkable restoration was observed for SOD activity (*p* < 0.01), CAT activity (*p* < 0.01), and GSH levels (*p* < 0.001) when compared to the control group receiving only MPTP. It was also observed that Til 10 + Lis was found more effective than Til 20 + Lis and levodopa to improve GSH level.

### 3.5. Effect of Co-Treatment of Til and Lis on TBARS

**Figure 4:**
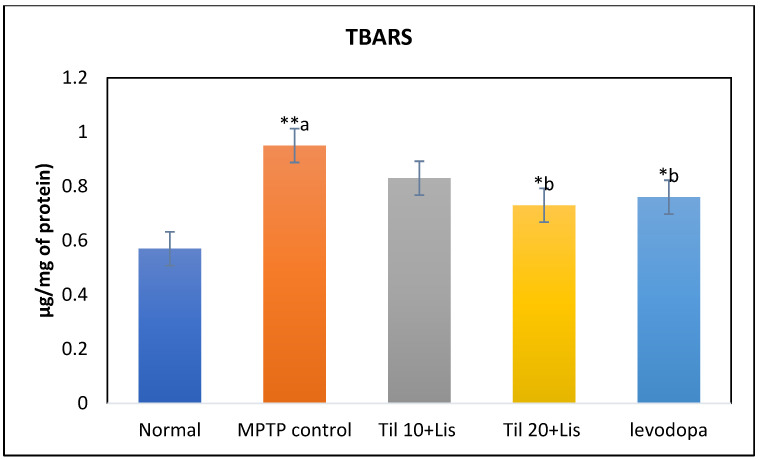
Effect of Til and Lis co-treatment on TBARS levels. The statistical significance was determined using a threshold of *p*-value (* *p* < 0.05; ** *p* < 0.01). “a”—normal vs. MPTP control; “b”—MPTP control vs. treatment groups. Data are expressed as the mean ± S.E.M. (*n* = 6). (F value 3.06.).

A significant reduction in TBARS levels was observed in the group treated with a co-treatment of Til and Lis, compared to the group treated with MPTP (*p* < 0.05), as demonstrated in Figure 4. This suggests that lipid peroxidation was effectively mitigated by the combined treatment, as indicated by the decreased formation of TBARS. These results imply that both Til and Lis might possess antioxidative properties that played a crucial role in their potential to provide protection against damage induced by oxidative stress.

### 3.6. Effect of Co-Treatment of Til and Lis on TNF-α, IL-1β and NF-κB

**Figure 5:**
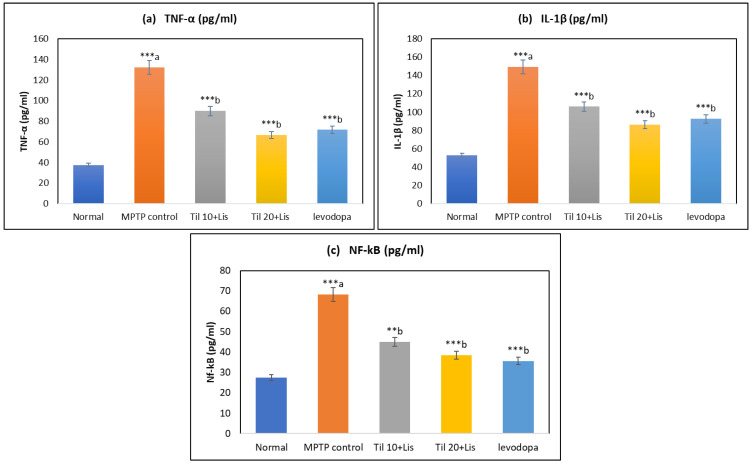
Effect of Til and Lis co-treatment on (**a**) TNF-α, (**b**) IL-1β, and (**c**) NF-κB levels. The statistical significance was determined using a threshold of *p*-value (** *p* < 0.01; *** *p* < 0.001). “a”—normal vs. MPTP control; “b”—MPTP control vs. treatment groups. Data are expressed as the mean ± S.E.M. (*n* = 6). (F value of TNF-α—58.73; IL-1β—33.84; NF-κB—37.634.).

It was found in the research that the MPTP control group had significantly higher levels of TNF-α and IL-1β compared to the normal group (*p* < 0.001). TNF-α and IL-1β levels were significantly reduced by the Til and Lis treatments compared to the MPTP group (*p* < 0.001). These findings demonstrated that the pro-inflammatory response was reduced, and cytokine levels were lowered by the co-treatment. Insights into the impact of treatments on inflammation and immune responses were provided by NF-κB levels analysis across different experimental conditions. It was indicated by the data that the potential to lessen inflammation was possessed by the combination of Til and Lis, particularly at higher doses, highlighting their potential as therapeutic agents. It was implied that anti-inflammatory properties might be possessed by Til and Lis through their capacity to modulate cytokine production. Notably, these effects of co-treatment were found to be comparable to those observed with levodopa treatment, as illustrated in Figure 5.

### 3.7. Effect of Co-Treatment of Til and Lis on Caspase 3 and BCL-2

**Figure 6:**
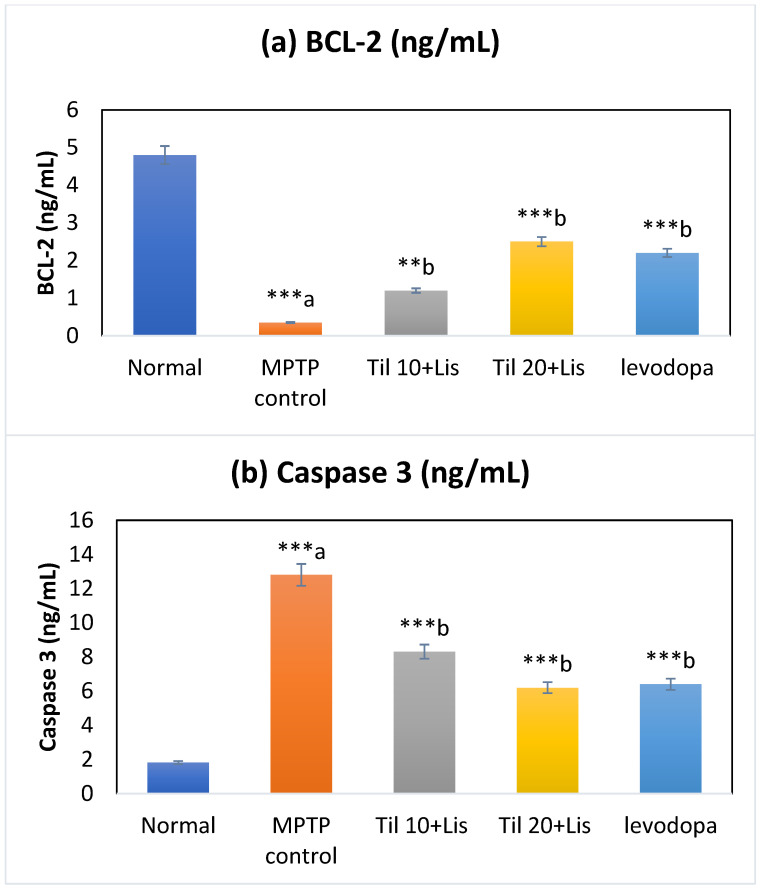
Effect of Til and Lis co-treatment on caspase 3 and BCL-2 levels. The statistical significance was determined using a threshold of *p*-value (** *p* < 0.01; *** *p* < 0.001). “a”—normal vs. MPTP control; “b”—MPTP control vs. treatment groups. Data are expressed as the mean ± S.E.M. (*n* = 6). (F value of caspase 3—49.03; BCL-2—126.71).

Figure 6 shows the statistically significant (*p* < 0.001) difference in Bcl-2 expression between the MPTP control and normal groups. The levels of cleaved caspase 3 were found to be elevated in the MPTP control group after the activation of pro-caspase 3 was induced by MPTP (*p* < 0.001). Caspase 3 levels were significantly altered by co-treatment compared to MPTP controls. It is noteworthy that Til 20+Lis showed efficacy on par with levodopa therapy.

### 3.8. Effect of Co-Treatment of Til and Lis on pAKt/Akt Expression Level

**Figure 7:**
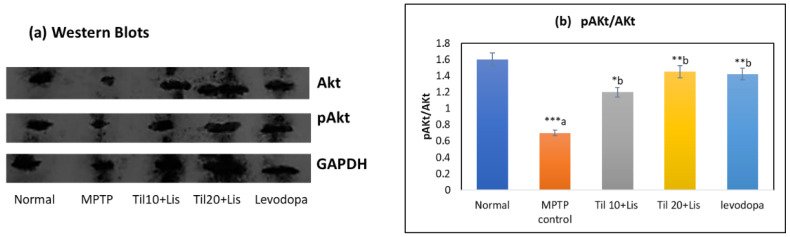
Western blot analysis of the protein level of p-Akt/Akt in the brain homogenates. (**a**) Western blotting; (**b**) p-Akt/Akt. The statistical significance was determined using a threshold of *p*-value (* *p* < 0.05; ** *p* < 0.01; *** *p* < 0.001). “a”—normal vs. MPTP control; “b”—MPTP control vs. treatment groups. Data are expressed as the mean ± S.E.M. (*n* = 6). (F value—6.50.).

A significant decrease in the pAkt/Akt ratio was demonstrated by the experimental results following MPTP treatment, indicating impaired Akt signaling when compared to the normal control group (*p* < 0.001), as depicted in Figure 7. However, when Til and Lis were administered together with MPTP, this reduction in the pAkt/Akt ratio was effectively alleviated (*p* < 0.01). This suggests a protective effect of these compounds on Akt activation. The findings suggest a potential positive influence of Til and Lis on the Akt signaling pathway in the MPTP model of neurodegenerative disorders. As a result, these compounds have the potential to act as modulators of Akt activity and may hold therapeutic implications for individuals affected by such conditions.

## 4. Discussion

PD is a complex neurological condition with both movement and non-movement symptoms. Its exact causes are unclear, hindering effective treatments. Research suggests brain inflammation and the PI3K/Akt pathway play key roles. We are studying how Til and Lis may work together to impact these factors in PD, seeking new treatment possibilities.

We investigated the combined administration of Til and Lis on behavioral measures on day 5 and day 11. MPTP-only treated group showed abnormal behaviors similar to PD symptoms. Til+Lis and levodopa treatments improved these behaviors, with Til 20+Lis showing the most promise. Levodopa was also effective. Further research should explore the mechanisms of these treatments. This aligns with a prior study on African herbal remedies protecting against PD in vitro [39]. In our research, we studied how combining Til and Lis affects activities such as pole climbing, holding time, and latent time in rats with MPTP-induced Parkinsonism. The results suggest that this combination can improve behavior in these rats, with the best outcomes seen with Til 20 and Lis. The rotarod test is commonly used to assess motor skills in rodents, including motor coordination and learning, especially in models with cerebellar deficits or PD [40,41,42]. Our research findings indicate that the co-administration of Til and Lis does indeed exert a noteworthy impact on rat motor coordination.

Oxidative stress targeting dopaminergic neurons is a primary driver of neurodegeneration in PD. Antioxidants, which counter ROS and free radicals, play a crucial role in preventing PD. The intracellular free-radical-generating agent MPTP induces oxidative stress and is implicated in PD pathogenesis, emphasizing its role in PD onset [43,44,45,46]. Key antioxidants such as CAT, SOD, and GSH play a crucial role in protecting cells from damage. In Parkinson’s disease, there is a significant decrease in these antioxidants, worsening the disease’s progression over time [47,48]. In a study conducted by Dongjie et al., the neuroprotective potential of Tongtian oral liquid, a Traditional Chinese Medicine, was explored within a zebrafish model of PD. The study elucidated the beneficial effects of this herbal remedy in mitigating the disease’s effects on neurodegeneration, highlighting its potential therapeutic value [49]. In our research, we investigated the antioxidant enzymes (GSH, SOD, and catalase) that combat harmful free radicals. In the TiI+Lis group, the levels of these enzymes were significantly higher compared to the MPTP control group. This finding suggests a promising defense against oxidative stress in the TiI+Lis group, with potential implications for managing conditions related to oxidative stress.

The relationship between TNF-α, IL-1β, NF-κB, and the development of PD’s complexity is confirmed. Neuroinflammation, fueled by TNF-α and IL-1β released by active glia, is crucial for PD’s progression. NF-κB, regulating stress-induced immune response, can amplify pro-inflammatory genes when triggered, leading to lasting inflammation and neuronal damage. This research explored the impact of co-treatment with Til and Lis on neuroinflammation in rats affected by PD due to MPTP exposure. The data revealed that the combined treatment notably decreased the pro-inflammatory cytokines, including IL-1β and TNF-α, in the brains of the affected rats. It also reduced the activity of NF-κB, a primary agent in inflammation. Our findings highlight that MPTP triggers intense neuroinflammation in rats, activating the TLR-4/NF-κB signaling pathway [50]. NF-κB is instrumental in driving inflammatory responses. When dormant, NF-κB is tied to IκB in the cell cytoplasm. However, when triggered, it detaches from IκB and moves to the cell nucleus, instigating the transcription of specific genes [51]. The combined treatment notably influenced these subsequent actions, depicted in flow diagram 1. Prior research indicates that Til curbs TRAF-6/NF-κB/p38-led neuroinflammation in activated BV2 microglia [22]. Moreover, Til significantly decreases swelling and the influx of leukocytes caused by tetradecanoylphorbol acetate [52]. Such anti-inflammatory and antioxidant qualities of Til could be the foundation of its neuroprotective effects. 

The significance of caspase 3 activation is of pronounced importance within the framework of PD pathology. This activation has the capacity to initiate apoptotic demise of neuronal cells and trigger the activation of microglial cells, thereby inciting inflammatory cascades. Consequently, the inhibition of caspase 3 activation holds the potential for a dual advantage within the cerebral milieu, potentially impeding the progression of PD [53]. Co-administration of both Til and Lis yielded a noticeable reduction in caspase 3 levels, as evidenced by a comparative analysis with the control cohort subjected to MPTP treatment.

**Scheme 3:**
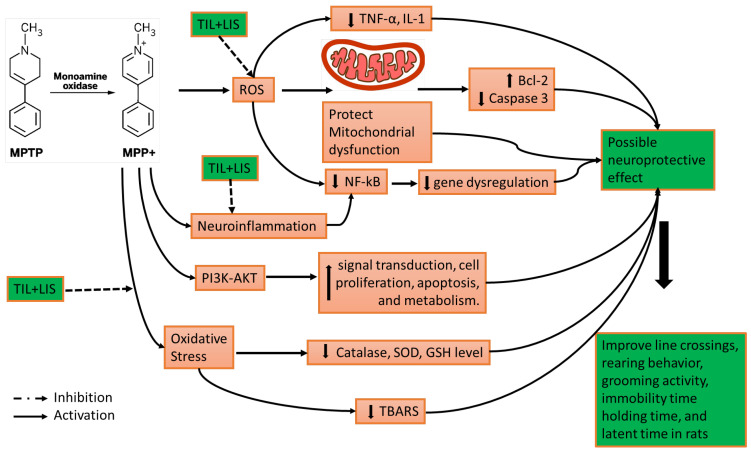
Possible mechanism of TIL+LIS in neuroprotection in PD.

Lis via 5-HT_2A_ receptors activates the Akt pathway; furthermore, the PI3K/Akt signaling pathway, critical for neuronal growth, proliferation, survival, and function, has been linked to preventing dopaminergic neuron loss caused by MPTP in PD mice [54]. This study assessed Akt and pAkt expression levels. Our findings demonstrated that MPTP administration substantially lowered the pAkt/Akt ratio compared to the controls. However, concurrent administration of Til and Lis effectively counteracted this decrease in the pAkt/Akt ratio. This suggests potential benefits of these compounds in mitigating or reversing MPTP-induced PD. Prior investigations also reported similar outcomes using different compounds such as ACT001, a derivative of parthenolide. ACT001 demonstrated synergy with low doses of L-DOPA, resulting in improved results in mouse models of MPTP-induced PD. These findings emphasize the potential advantages of combining phytoconstituents with a minimal dose of synthetic PD medication [55]. The possible mechanism of TIL+LIS is shown in Scheme 3.

This research suggests that the combination of Til and Lis demonstrates superior efficacy in the management of PD compared to the administration of levodopa. Levodopa monotherapy has been associated with the development of involuntary movements over an extended period of time. The selection of Til, a naturally occurring molecule possessing antioxidant and neuroprotective qualities, and Lis, a dopamine agonist, was made for the purpose of implementing this combined therapeutic approach. The available research indicates that the combination of these two elements presents a considerable opportunity to safeguard neurones responsible for dopamine production against degeneration in a rat model of PD. This combination offers a viable alternative to address the limitations associated with levodopa treatment. The method entails the suppression of neuroinflammation, the mitigation of apoptosis, and the facilitation of neuronal survival, presenting a potentially effective approach to slowing the progression of neurodegeneration in PD.

## 5. Conclusions

In summary, this research underscores the potential of combining Til with low doses of Lis to yield amplified benefits in alleviating PD. Through their synergistic interaction, these compounds present a supplementary avenue for PD treatment. This approach holds promise for ameliorating symptoms, slowing disease progression, and ultimately enhancing therapeutic outcomes. These findings underscore the significance of exploring innovative drug combinations to devise more efficacious management and treatment protocols for Parkinson’s disease. Remarkably, this study revealed Til’s ability to curtail neuroinflammation, a pivotal mechanism implicated in PD pathogenesis, as evidenced by the substantial reduction in pro-inflammatory factors observed in rat brain tissues treated with Til. Moreover, Til’s potential to inhibit apoptosis and bolster neuronal survival implies its role in preserving dopaminergic neurons critical for sustaining motor function in PD patients. In essence, our findings highlight the auspicious neuroprotective attributes of Til and its potential as a valuable augmentation to current therapeutic approaches in PD management. Further exploration through research and clinical investigations is warranted to fully elucidate Til’s therapeutic potential in PD and to optimize its integration alongside existing PD medications such as Lis by addressing limitations associated with prolonged Lis usage and offering fresh avenues for effective PD treatment.

## Figures and Tables

**Table 1:** The impact of co-treatment with Til and Lis on the behavior of rats in the open-field test was assessed in this study.

Groups	Line Crossing		Rearing		Grooming		Immobility	
	Day 5	Day 11	Day 5	Day 11	Day 5	Day 11	Day 5	Day 11
Normal	141.67 ± 8.05	150.83 ± 6.16	15.67 ± 1.51	17.67 ± 1.23	20.33 ± 3.15	25.33 ± 1.87	11.33 ± 1.90	18.83 ± 2.10
MPTP control	23.50 ± 4.98 ^a^***	36.17 ± 3.07 ^a^***	3.10 ± 0.33 ^a^***	3.83 ± 0.65 ^a^***	5.33 ± 1.09 ^a^***	8.33 ± 1.09	194.33 ± 2.71 ^a^***	215.67 ± 4.01 ^a^***
Til 10+Lis	39.67 ± 3.95	46.67 ± 2.125	4.71 ± 0.78	6.83 ± 0.91	7.67 ± 0.80	17.33 ± 1.34	165.33 ± 7.67 ^b^**	155.67 ± 6.44 ^b^***
Til 20+Lis	54.17 ± 6.38 ^b^**	72.83 ± 4.6 ^b^***	8.17 ± 0.87 ^b^**	9.17 ± 1.51 ^b^*	11.67 ± 1.46	20 ± 1.7 ^b^*	129.67 ± 7.07 ^b^***	122.83 ± 7.95 ^b^***
Levodopa	45.83 ± 4.15	63.67 ± 4.81 ^b^**	6.50 ± 0.92	9.17 ± 1.2 ^b^*	9.83 ± 1.45	17.33 ± 2.04 ^b^*	136.17 ± 4.58 ^b^***	132.67 ± 8.03 ^b^***

Four behavioral parameters were measured: line crossing (day 5 F value—107.29; day 11 F value—107.95), rearing (day 5 F value—26.034; day 11 F value—20.212), grooming (day 5 F value—10.45; day 11 F value—14.16), and immobility time (day 5 F value—175.27; day 11 F value—136.49). To determine statistical significance, a *p*-value threshold (* *p* < 0.05; ** *p* < 0.01; *** *p* < 0.001) was utilized. Comparisons were made between different groups: “a” represents normal vs. MPTP control while “b” stands for MPTP control vs. treatment groups. The data presented are expressed as mean ± S.E.M., with *n* = 6 samples used for analysis.

## Data Availability

All data generated or analyzed during this study are included in this published article.
